# Oral Contraceptive Use and Increased Risk of Stroke: A Dose–Response Meta-Analysis of Observational Studies

**DOI:** 10.3389/fneur.2019.00993

**Published:** 2019-09-23

**Authors:** Feng Li, Lin Zhu, Jie Zhang, Hongye He, Yueqi Qin, Yuan Cheng, Zongyi Xie

**Affiliations:** ^1^Department of Neurosurgery, The Second Affiliated Hospital, Chongqing Medical University, Chongqing, China; ^2^College of Basic Medicine, Chongqing Medical University, Chongqing, China; ^3^Clinical Medical College, Southwest Jiaotong University, Chengdu, China; ^4^Department of Neurosurgery, Children's Hospital, Chongqing Medical University, Chongqing, China

**Keywords:** oral contraceptive, estrogen dosage, duration, stroke, dose–response, meta-analysis

## Abstract

**Background:** Oral contraceptive (OCP) use might increase the risk of stroke in women. We examined a possible dose–response relation between OCP use and the risk of stroke in young and middle-aged women.

**Methods:** A retrieval of PubMed and EMBASE databases was performed. We selected observational studies that reported odds ratios (ORs) with 95% confidence intervals (CIs) for the risk of stroke in OCP users. A two-stage dose–response analysis was conducted using the random-effects model and the restricted spline model.

**Results:** A total of 6 cohort studies and 12 case–control studies were included, which involved 2,143,174 participants and 11,661 cases of stroke including ischemic stroke (IS), hemorrhagic stroke (HS), and stroke of unknown origin. The pooled ORs of total stroke were 1.19 (95% CI, 1.16–1.23) for every 10-μg increment in estrogen dosage, 1.20 (95% CI, 1.05–1.37) for every 5-years increment in duration of OCP use, and 0.82 (95% CI, 0.68–0.98) for every 5-years increment in duration of OCP cessation. The ORs of IS were 1.20 (95% CI, 1.17–1.22) in estrogen dosage, 1.24 (95% CI, 1.04–1.49) in duration of OCP use, and 0.78 (95% CI, 0.67–0.92) in duration of OCP cessation. The ORs of HS were 1.10 (95% CI, 1.04–1.16) in estrogen dosage, 1.13 (95% CI, 0.93–1.36) in duration of OCPs, and 0.71 (95% CI, 0.55–0.92) in duration of OCP cessation. The pooled ORs of total stroke from prospective studies (1.12; 95% CI, 1.01–1.24) were lower than those from retrospective studies (1.30; 95% CI, 1.01–1.67).

**Conclusions:** The higher estrogen dosage significantly increased the risks of total stroke, IS, and HS, respectively. The longer duration of OCP use significantly increased the risks of total stroke and IS, but its effects on HS risk were marginal. The longer duration of OCP cessation significantly decreased the risks of total stroke, IS, and HS, respectively. These findings affirm the contribution of estrogen dose and duration of OCP use to the increased risk of stroke, which may be critical for the instruction of OCP use and the prevention and management of cerebrovascular diseases.

## Introduction

Stroke is a cerebrovascular disease with high mortality and morbidity (1). According to the heart disease and stroke statistics update 2018 from the American Heart Association (AHA), females had higher lifetime incidence and mortality of stroke than males in the United States (2). In 1969, Vessey et al. first reported that oral contraceptives (OCPs) were the cause of venous thromboembolism and cerebral thrombosis in women (3). Subsequently, numerous observational studies have been conducted to assess the association between OCP use and stroke incidence.

To date, it remains controversial whether women who are taking OCPs are at an increased risk of stroke (4–10). A recent meta-analysis indicated that the risk of first-ever ischemic stroke (IS) was increased significantly in females currently taking OCPs (11). Similarly, another meta-analysis study showed that current OCP use led to a small increase in hemorrhagic stroke (HS) incidence (12). Although Roach et al. found that the OCP users were not at increased risk of IS, they stratified their meta-analysis according to estrogen dose and found that there was significant risk of stroke in women taking combined OCPs with estrogen doses more than 50 μg (13). However, the high heterogeneity of data in these studies devalued the reliability of conclusions. Thus, we further explored the dose–response relation between OCP use and the risk of stroke in detail.

In the present study, we performed a meta-analysis to assess the dose-dependent relation between OCP use and the risk of stroke including total stroke, IS, and HS. Analyses were stratified according to estrogen dosage in combined OCPs, duration of OCP use, and duration of OCP cessation.

## Methods

### Literature Search and Study Selection

This systematic review and meta-analysis was conducted in accordance with PRISMA guidelines (14). A systematic literature search was performed through PubMed and EMBASE databases from their inception to September 2018. The detailed search strategy was recorded in Supplemental Data. We also searched Cochrane Library, in which the results were few and almost duplicated those found in PubMed and EMBASE. The reference lists of retrieved articles were checked manually to prevent literature omission. If necessary, extra information or data were obtained by contacting the original authors. Observational studies were included in the meta-analysis if they met the following criteria: (1) Participants: patients who were stroke-free at the beginning of study. (2) Exposure: there were quantitative parameters of OCPs including estrogen doses (μg), duration of OCP use (years), or duration of OCP cessation (years). (3) Outcome: adjusted odds ratios (ORs) with 95% confidence intervals (CIs) were assessed for the risk of stroke (total stroke, IS, or HS) with parameters of OCPs. (4) Additional data: number of stroke cases and controls, total subjects or person-years. (5) Study design: retrospective cohort, prospective cohort, nest case–control, or case–control study. (6) Language: articles were published in English.

### Data Extraction and Quality Assessment

One researcher conducted data extraction in a standard data-collection form, and a second investigator checked for accuracy. Information or data were recorded as follows: first author, publication year, study location, sample size in cohort studies, number of stroke cases and controls in case–control studies, status of OCP use, assessment methods of OCP use and stroke incidents, the maximally adjusted effect values with the corresponding 95% CIs compared to OCPs non-users and the effect values compared to current OCP users in OCP cessation, and adjustment factors of the multivariate analysis.

Literature quality was evaluated by two investigators independently using the Newcastle-Ottawa quality assessment scale (NOS) (15). The overall risk of bias was assessed using three aspects of scale: selection of study subjects, comparability of groups, and ascertainment of exposure and outcomes. The quality of studies was ranked as low (below three stars), moderate (4–6 stars), and high (7–9 stars). Nine stars were the highest score if the study met all assessment criteria. Any discrepancy was resolved by further discussion between two investigators.

### Statistical Analyses

In this meta-analysis, the risk was assessed by OR in the case–control study and relative risk (RR) in the cohort study with the corresponding CIs. Because of absolutely low incidence of stroke in women, especially young women, the OR was considered to be approximate to RR. Therefore, OR was used to report the risk of stroke with OCP use in all included studies. We stratified the dose effect of OCP use by the following three quantitative parameters: estrogen dosage, duration of OCP use, and duration of OCP cessation. Due to inconsistent cut-off points of OCP categories across all included studies, we summarized pooled ORs with 95% CIs for “unit increment,” namely, 10-μg increment in estrogen doses, 5-years increment in duration of OCP use, and 5-years increment in duration of OCP cessation. The between-study heterogeneity was tested by (1) *Q* statistics with *P* < 0.10 as significance of heterogeneity and (2) *I*^2^ statistic (16) with the following three significances of heterogeneity in general: *I*^2^ < 50% as low heterogeneity; 50% ≤ *I*^2^ < 75% as moderate heterogeneity; *I*^2^ ≥ 75% as high heterogeneity (17).

The two-stage dose–response meta-analysis was performed using generalized least-squares regression trend estimation as described by Orsini et al. (18) and Greenland and Longnecker (19). We calculated stroke risks for every “unit increment” from linear trend in each quantitative parameter of OCP use followed by a random-effects model to obtain pooled ORs and 95% CIs (20). We also examined the potential non-linear relationship on the basis of ORs of each quantitative OCPs parameter using restricted cubic splines with four knots at percentiles 5, 35, 65, and 95% of the distribution (21, 22). The three-knot model was applied to assess the risk of HS and estrogen dosage of OCPs. ${Chi}_{model}^{2}$ and *P*_*model*_ were utilized to check the suitability of model. The linear or non-linear curve coincidence with these associations was estimated by *P*_*non*−*linearity*._The value of *P*_*non*−*linearity*_ was obtained by testing the null hypothesis that the coefficient of the solid line is equal to 0.21 A *P*_*non*−*linearity*_ < 0.05 was considered statistically significant, suggesting a non-linear tendency. Person-years was acquired directly in most studies or estimated by our own calculation. In addition, the midpoint of lower and upper boundaries was designated as the dose of each quantitative parameter category if there was no mean or median level reported in the study. If the highest category was half open interval, the dose of this OCP parameter category was set at 1.2 times of the upper boundary. The lower boundary was set to zero if the lowest category was half open interval.

Apart from the primary dose–response analyses, the study-level subgroup analysis was stratified by region, study design type, OCP status, and the adjustment of potential confounders [smoking, hypertension, diabetes, alcohol, body mass index (BMI), and education]. The *P*_*interaction*_ among subgroups was tested by meta-regression (23). To examine stability of results and find sources of heterogeneity, the following three methods of sensitive analyses were carried out: (1) ignoring a single study in turn; (2) calculating ORs by both effect model and fixed model; and (3) adding extra eligibility criterion: only included studies “confounders adjust for smoking, hypertension, and diabetes,” “ORs refer to OCPs non-users,” and “women of age less than 18 years,” and excluded studies “fatal stroke” and “women of age more than 50 years.” The publication bias was investigated by Begg's test (24) and Egger's test (25). STATA version 12.0 (StataCorp, College Station, TX) was used for all analyses. A value of *P* < 0.05 was considered significant, except where otherwise specified.

## Results

### Study Level Characteristics

A total of 4,170 and 1,008 potential eligible articles were identified from PubMed database and EMBASE database, respectively. The whole literature searching process was presented in Supplemental Figure 1. During full-article screening, the primary cause of study ineligibility was the lack of quantitative data of OCP use, and more detailed reasons of exclusion in full-article view are shown in Supplemental Table 1. In the end, 18 publications including 6 cohort studies and 12 case–control studies were included for the final data analysis. The characteristics of all selected articles are depicted in Supplemental Table 2. Among them, 10 studies were conducted in Europe9 (26–35), 3 studies in Asia (10, 36, 37), 1 study in Oceania (38), 1 study in North America (39), and 2 transnational studies among Africa, America, Asia, and Europe (40, 41). The age of the subjects in all of 18 studies ranged from 15 to 79 years old, in which 12 studies were conducted in a population under 50 years old. The mean follow-up time of cohort studies ranged from 2.9 to 18.6 years. The total of 2,143,174 participants and 11,661 stroke cases were included in this meta-analysis. These original studies assessed OCP use by various methods, in which questionnaires or face-to-face interview was the most common. The investigators independently validated self-reported use of OCPs through reviewing pharmacy records. Medical records or national medical statistics were also used in other studies (26, 29, 34). Stroke diagnosis was confirmed through various methods, of which most was medical records and the rest was national registry of patients or death (9, 10, 28–30). With respect to literature quality score, almost all literature were high quality (score ≥ 7) except one with moderate quality (score = 6) (Supplemental Table 3).

### OCP Use and Risk of Total Stroke

**Figure 1:**
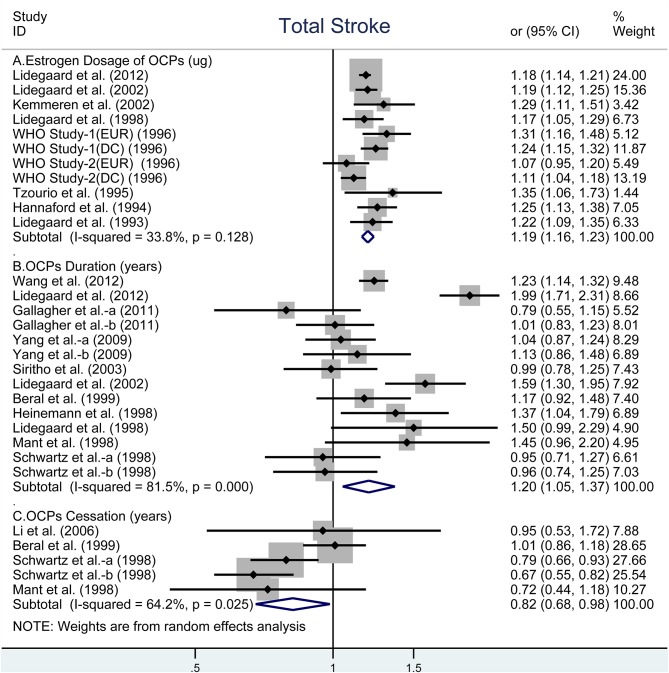
Forest plot of studies examining the association between OCP use [**(A)** estrogen dosage of OCPs, **(B)** OCPs duration, **(C)** OCPs cessation] and risk of total stroke.

The risk of total stroke was significantly increased with OR of 1.19 (95% CI, 1.16–1.23) for every 10-μg estrogen increment in estrogen dosage (26, 28–31, 33, 35, 40, 41) and OR of 1.20 (95% CI, 1.05–1.37) for every 5-years increment in duration of OCP use (9, 10, 27–29, 32–34, 37–39), but was decreased with OR of 0.82 (95% CI, 0.68–0.98) for every 5-years increment in duration of OCP cessation (27, 34, 36, 39). Heterogeneity existed in total stroke risk estimates across all selected studies for estrogen dosage (*I*^2^ = 33.8%, *P*_*heterogeneity*_ = 0.128, Figure 1A), for duration of OCP use (*I*^2^ = 81.5%, *P*_*heterogeneity*_ < 0.001, Figure 1B), and for duration of OCP cessation (*I*^2^ = 64.2%, *P*_*heterogeneity*_ = 0.025, Figure 1C).

### OCP Use and Risk of Ischemic Stroke

**Figure 2:**
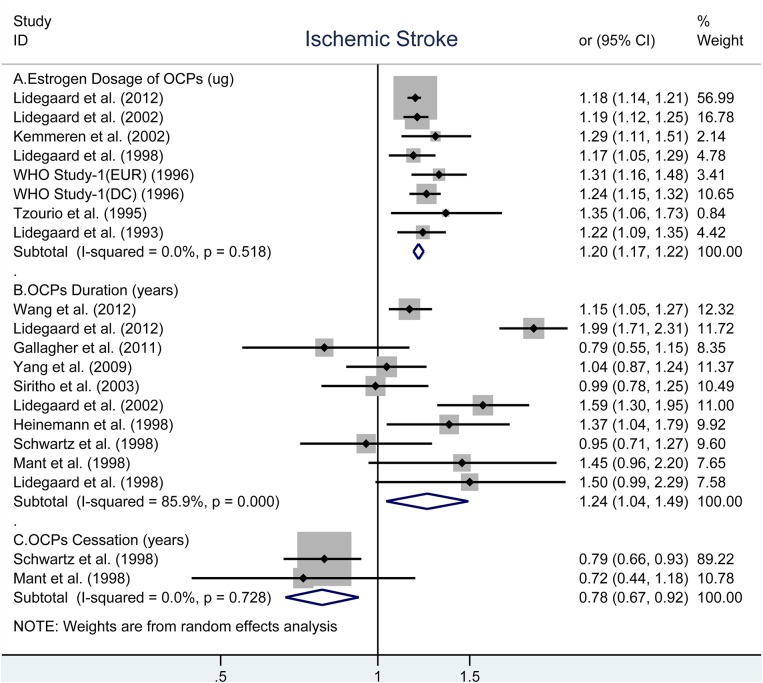
Forest plot of studies examining the association between OCP use [**(A)** estrogen dosage of OCPs, **(B)** OCPs duration, **(C)** OCPs cessation] and risk of ischemic stroke.

Similar to total stroke, the analyses showed that the obviously increased risk of IS associated with every 10-μg increment in estrogen dosage (OR, 1.20; 95% CI, 1.17–1.22, Figure 2A) (28–31, 33, 35, 40) and every 5-years increment in duration of OCP use (OR, 1.24; 95% CI: 1.04–1.49, Figure 2B) (9, 10, 27–29, 32, 33, 37–39), but decreased risk for every 5-years increment in duration of OCP cessation (OR, 0.78; 95% CI: 0.67–0.92) (27, 39). There were no evidence of heterogeneity across all selected studies in estrogen dosage (*I*^2^ = 0%, *P*_*heterogeneity*_ = 0.518) and duration of OCP cessation (*I*^2^ = 0%, *P*_*heterogeneity*_ = 0.728, Figure 2C). However, high heterogeneity was observed between duration of OCP use and risk of IS (*I*^2^ = 85.9%, *P*_*heterogeneity*_ < 0.001).

### OCP Use and Risk of HS

**Figure 3:**
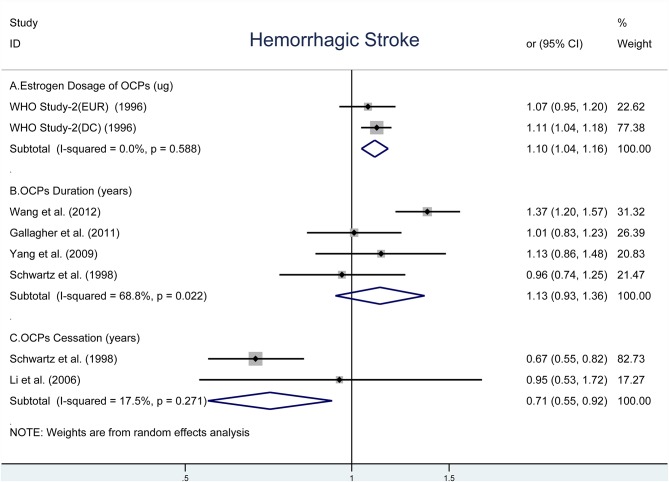
Forest plot of studies examining the association between OCP use [**(A)** estrogen dosage of OCPs, **(B)** OCPs duration, **(C)** OCPs cessation] and risk of hemorrhagic stroke.

As shown in Figure 3, there was an elevated risk of HS for each increase in 10-μg estrogen dosage (OR, 1.10; 95% CI, 1.04–1.16) without heterogeneity (*I*^2^ = 0%, *P*_*heterogeneity*_ = 0.588) (Figure 3A) (42). The pooled OR was 1.13 (95% CI, 0.93–1.36) for each increase in 5-years duration of OCP use with moderate heterogeneity (*I*^2^ = 68.8%, *P*_*heterogeneity*_ = 0.022, Figure 3B) (9, 10, 37, 39). However, the risk of HS reduced significantly for each increase in 5-years duration of OCP cessation (OR, 0.71; 95% CI, 0.55–0.92) with estimated low heterogeneity (*I*^2^ = 17.5%, *P*_*heterogeneity*_ = 0.271, Figure 3C) (36, 40).

### Linear Tendency Analyses

**Figure 4:**
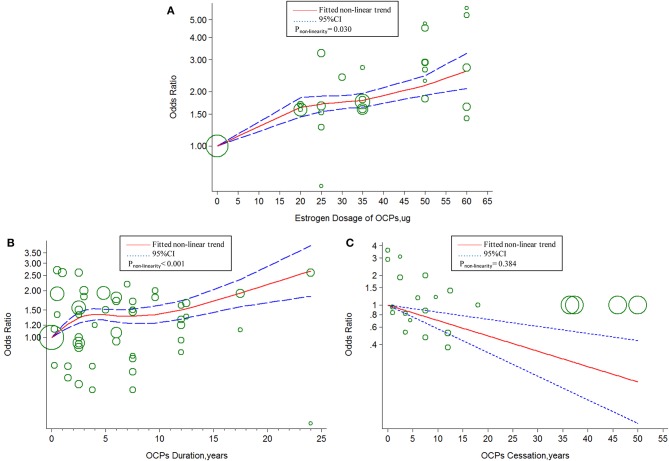
Dose-response relationship between OCPs [**(A)** estrogen dosage of OCPs, **(B)** OCPs duration, **(C)** OCPs cessation] and risk of total stroke. The circles represent the ORs in each individual study with the circular size reflecting the weight of corresponding study. OCP, oral contraceptives.

**Figure 5:**
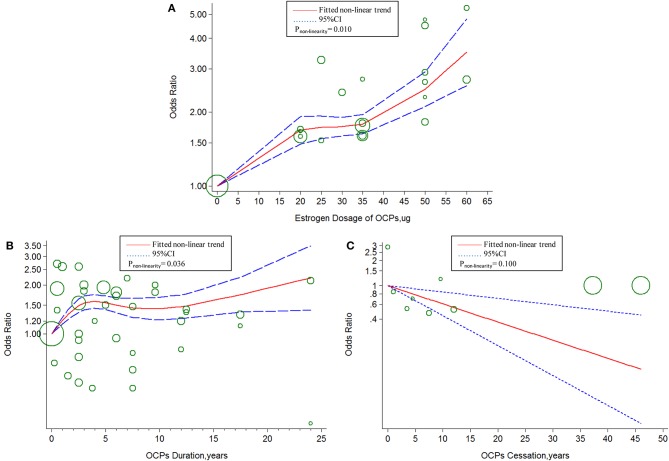
Dose–response relationship between OCPs [**(A)** estrogen dosage of OCPs, **(B)** OCPs duration, **(C)** OCPs cessation] and risk of ischemic stroke. The circles represent the ORs in each individual study with the circular size reflecting the weight of corresponding study. OCP, oral contraceptives.

**Figure 6:**
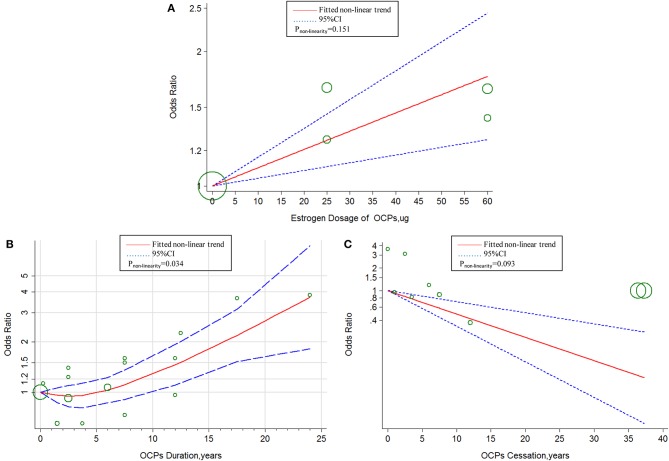
Dose–response relationship between OCPs [**(A)** estrogen dosage of OCPs, **(B)** OCPs duration, **(C)** OCPs cessation] and risk of hemorrhagic stroke. The circles represent the ORs in each individual study with the circular size reflecting the weight of corresponding study; OCP, oral contraceptives.

The linear tendency of this dose–response analysis was evaluated by the restricted cubic spline model. With respect to total stroke, evidences of non-linear association with estrogen dosage (*P*_*non*−*linearity*_ = 0.030; ${Chi}_{model}^{2}$ = 268.23; *P*_*model*_ < 0.001; Figure 4A) and duration of OCP use (*P*_*non*−*linearity*_ < 0.001; ${Chi}_{model}^{2}$ = 96.55; *P*_*model*_ < 0.001; Figure 4B) were identified, but a linear relationship was found with duration of OCP cessation (*P*_*non*−*linearity*_ = 0.384; ${Chi}_{model}^{2}$ = 15.16; *P*_*model*_ = 0.002; Figure 4C). For IS, there was a non-linear association with estrogen dosage (*P*_*non*−*linearity*_ = 0.010; ${Chi}_{model}^{2}$ = 249.51; *P*_*model*_ < 0.001; Figure 5A) or duration of OCP use (*P*_*non*−*linearity*_ = 0.036; ${Chi}_{model}^{2}$ = 6.77; *P*_*model*_ = 0.079; Figure 5B), but a linear association with duration of OCP cessation (*P*_*non*−*linearity*_ = 0.099; ${Chi}_{model}^{2}$ = 13.76; *P*_*model*_ = 0.003; Figure 5C). For HS, the linear relationship was found with estrogen dosage (*P*_*non*−*linearity*_ = 0.151; ${Chi}_{model}^{2}$ = 13.59; *P*_*model*_ = 0.001; Figure 6A), but a non-linear relationship was found with duration of OCP use (*P*_*non*−*linearity*_ = 0.034; ${Chi}_{model}^{2}$ = 19.00; *P*_*model*_ < 0.001; Figure 6B). Similarly, linear evidence was found for risk of HS with duration of OCP cessation (*P*_*non*−*linearity*_ = 0.093; ${Chi}_{model}^{2}$ = 18.72; *P*_*model*_ < 0.001; Figure 6C).

### Subgroup Analyses and Sensitivity Analyses

Subgroup analyses were conducted to explore possible sources of heterogeneity using a meta-regression model (Supplemental Table 4). While the significantly increased risks of total stroke and IS were noted in “developed counties,” “case–control studies,” “current OCPs usage,” “adjustment for smoking and hypertension,” and “adjustment for family stroke-history,” the marginally increased risks of total stroke and IS were observed in the subgroups opposite to those aforementioned. Subgroup analyses regarding HS or OCP cessation failed to be completed due to the limited number of relevant studies. The pooled ORs were virtually identical when calculated using either fixed or random-effects model. The omission of each single study did not affect overall results. When we added some extra inclusion or exclusion criteria, there were no significant changes in the results (Supplemental Table 5).

### Publication Bias

No evidence of publication bias was revealed by Begg's funnel plot and Egger's regression test (all *P* > 0.1).

## Discussion

In the present study, a two-stage dose–response analysis revealed the dose dependence between OCP use and the risk of stroke. Our findings indicated that (1) the increased estrogen dosage and duration of OCP use were associated with the increased risk of stroke in women, and the longer duration of OCP cessation was related to less risk of stroke; (2) there was only a marginal significance between duration of OCP use and increased HS risk; (3) there was an approximate linear association of estrogen dosage or OCP cessation but a non-linear association of duration of OCP use with the risk of stroke.

A large number of studies previously investigated the relationship of OCP status and cardiovascular diseases (CVDs), such as myocardial infarction (42–44), venous thrombosis (45, 46), and hypertension (47). However, only one study systematically analyzed the dose effect between duration of OCP use and risk of hypertension (48). To the best of our knowledge, the current study was the first dose–response meta-analysis between OCP use and the risk of stroke.

The two latest correlative meta-analyses explored the association between OCP use status and IS (11, 13). One meta-analysis revealed that current use of OCPs was associated with an increased risk of IS (11), and the risk of IS among current OCP users was increased along with increase in estrogen dose of different categories, which were consistent with our results. The other one showed that there was no association between current OCP use and IS, but an apparent risk of IS was observed if OCPs contained high doses of estrogen (≥50 μg) or second-generation progestin (13). In the current study, we observed that there was ~20% increase in the risks of IS and total stroke with every 10-μg estrogen or 5-years duration increment of OCP use. In contrast, there was about a 20% decreased risk with every 5-years increment in OCP cessation. The results confirmed the persistent detrimental effects of high doses of estrogen and length of OCP use on stroke incidence in women. The non-linear curve of association between duration of OCP use and total stroke or IS suggested the stroke risk ascending within 5 years of OCP use, becoming relatively stable during 5–10 years, and rising again at a lower slope after 10 years. The poor adherence to long-term OCP medication and the small number of long-term studies may contribute to the non-linear result.

Another meta-analysis summarized a small increased risk of HS in relation to current OCP use, which was consistent with our results (12). We demonstrated that a 1.1-fold increased risk of HS was associated with every 10-μg increment in estrogen dose, whereas 29% of decreased risk of HS was related to every 5-years increment in OCP cessation. The non-linear curve of association between risk of HS and duration of OCP use showed a stable HS risk within 5 years and gradually increased risk after 5 years. The possible explanation could be antagonism of non-identified protective factors in the beginning years of OCP use. Therefore, it is recommended that OCPs should not be taken more than 5 years for women with high risk of hemorrhage stroke.

Compared to high heterogeneity reported in previous meta-analyses, most of our results showed apparently lower heterogeneity. The discrepant results may be due to different estrogen dosage or progestin types in OCPs, and confounders, such as CVD risk factors. Nevertheless, a substantial heterogeneity was still found in risk of stroke with duration of OCP use. Thus, we further performed a series of subgroup analyses and observed a positive relationship between risk of total stroke and estrogen dosage in both developing countries and developed countries. However, the dose dependence relation between the risk of total stroke and OCP use was only significant in developed but not developing countries. Given merely two studies from developing countries with limited participants, the lack of statistic power may be responsible for the insignificance. Other possible reasons could be more confounders and poor hospital conditions for stroke diagnosis. Regarding the type of study design, both retrospective studies and prospective studies consistently demonstrated increased risk of stroke with increments in estrogen dosage and duration of OCP use, and the pooled OR of retrospective studies was higher than those of prospective studies. The subgroup analysis suggested that the risk of stroke related to duration of OCP use was likely overestimated by reason of the case–control study's methodology disadvantage with more bias of selection and recall than the cohort study. With respect to the status of OCP use, our results demonstrated that significantly increased risk of stroke only existed in current OCP users, which was consistent with the results of the majority of epidemiological studies (9, 10, 27, 38, 39) and meta-analyses (11, 12) published previously.

In addition, we carried out stratified analyses with some confounders adjusted. The results showed that the risk of stroke was slightly lower after these confounders were adjusted than those failed to be adjusted. There was strong evidence that incidence of stroke in subjects younger than 45 years old who smoked more than 20 cigarettes per day was at 5.04-fold higher compared to non-smokers (49). A progressive and linear relationship between incidence of stroke and BMI was shown in women in an Asian study (50). Another study also reported a linear association between risk of stroke and hypertension (48). Thus, these risk factors may be potential confounders that contribute to the heterogeneity and exacerbate the stroke risk in OCP users. It suggests that OCPs should not be the optimal method of birth control in women, with risk factors including smoking, drinking, obesity, hypertension, and diabetes. Especially for women who have been attacked by CVDs, they are advised to discontinue OCPs as soon as possible. Due to deficient available data on association between OCP use and risk of stroke in smokers vs. non-smokers, more studies are needed to examine the possibility of effect modification by smoking in the future.

The intrinsic biological mechanisms underlying the increased risk of stroke by OCPs are far from clear. Previous studies indicated that estrogen exerted potential detrimental effects through accelerating vascular endothelial cell growth and inhibiting proliferation of vascular smooth muscle cells via transcriptional regulation on relevant genes, which eventually resulted in a thickened and less elastic wall of blood vessels (37, 51, 52). Several studies also suggested that the use of OCPs was positively related to hypertension (47, 48) and dyslipidemia (53). Therefore, it was highly possible that OCPs accounted for alteration of vascular wall, blood pressure, and lipid metabolism, which collectively contributed to the increased stroke incidence.

There were some limitations in our study. First, all the studies included in the current meta-analysis were published more than 5 years ago, but the most combined OCPs in recent years have become OCPs of low-dose estrogen. Thus, the lack of latest research might weaken the risk estimate of estrogen. Second, all studies were observational studies that were vulnerable to the impact of potential confounders. The majority of case–control studies could especially enlarge the biases of recall, interview, and selection. Third, the limited relevant studies could account for the lack of statistical power to reveal the significant dose dependence relation between the duration of OCP use and the increased risk of HS. Fourth, we only included English literature, which also generated some bias in our results. At last, even though subgroup and sensitive analyses have been conducted to check origin of heterogeneity, the heterogeneity across studies was undeniable.

## Conclusions

The higher estrogen dosage was significantly associated with the increased risk of total stroke, IS, and HS, respectively. The longer duration of OCP use significantly contributed to the increased risk of total stroke and IS, while its effects on HS risk was marginal. The longer duration of OCP cessation, the less the risk of total stroke, IS, and HS. These findings confirmed the dose dependence between OCP use and stroke incidence, which may be used as reference for the use of OCPs and prevention and management of cerebrovascular diseases. Future studies are warranted to explore the potential underlying mechanisms of increased brain vulnerability to stroke by OCPs.

## Data Availability Statement

All datasets generated for this study are included in the manuscript/Supplementary Files.

## Author Contributions

ZX and FL: conception and design of the study. LZ and JZ: literature retrieval, study selection, and data extraction. HH and YQ: statistical analyses. YC: quality evaluation. FL: interpretation of the data and drafting of the initial manuscript. ZX: critical revision and comment for important intellectual content. All authors reviewed and approved the final manuscript. All authors were appreciative of Lei Huang, M.D., from the Department of Neurosurgery, School of Medicine, Loma Linda University, USA, for revising the manuscript.

### Conflict of Interest

The authors declare that the research was conducted in the absence of any commercial or financial relationships that could be construed as a potential conflict of interest.

## Abbreviations

OCPs: oral contraceptives
CIs: confidence intervals
ORs: odds ratios
IS: ischemic stroke
HS: hemorrhagic stroke.
