# Supplementary material for: Oral Contraceptive Use and Increased Risk of Stroke: A Dose–Response Meta-Analysis of Observational Studies
Source: Front Neurol. 2019 Sep 23;10:993. doi: 10.3389/fneur.2019.00993 (PMC6767325; doi:10.3389/fneur.2019.00993)
Supplement: Supplementary file 1 [file Data_Sheet_1.docx]

Supplementary Material

**Supplemental List 1 Search Strategy**

**PubMed (n=4170)**

#1: Oral contraceptive [Title/Abstract]) OR Birth control pill [Title/Abstract]

#2: "Contraceptives, Oral"[Mesh]

#3: #1 OR #2

#4: Stroke [Title/Abstract]

#5: "Stroke"[Mesh]

#6: #4 OR #5

#7: Intracranial hemorrhage [Title/Abstract]

#8: "Intracranial Hemorrhages"[Mesh]

#9: #7 OR #8

#10: Cerebral hemorrhage [Title/Abstract]

#11: "Cerebral Hemorrhage""[Mesh]"

#12: #10 OR #11

#13: Subarachnoid hemorrhage [Title/Abstract]

#14: "Subarachnoid Hemorrhage" [Mesh]

#15: #13 OR #14

#16: ((Intraparenchymal hemorrhage [Title/Abstract]) OR Brain hemorrhage [Title/Abstract]) OR Intracerebral hemorrhage [Title/Abstract]

#17: Cerebral infarction [Title/Abstract]

#18: "Cerebral Infarction"[Mesh]

#19: #17 OR #18

#20: (Brain ischemia [Title/Abstract]) OR Cerebral ischemia [Title/Abstract]

#21: "Brain Ischemia"[Mesh]

#22: #20 OR #21

#23: Cardiovascular disease [Title/Abstract]

#24: "Cardiovascular Diseases"[Mesh]

#25: #23 OR #24

#26: (Cerebrovascular disorder [Title/Abstract]) OR Cerebrovascular disease [Title/Abstract]"

#27: "Cerebrovascular Disorders"[Mesh]

#28: #26 OR #27

#29: #6 OR #9 OR #12 OR #15 OR #16 OR #19 OR #22 OR #25 OR #28

#30: #3 AND #29

#31: (Review [Publication Type]) OR Letter [Publication Type]

#32: #30 NOT #31

**EMBASE (n=1008)**

#1: oral contraceptive: ab,ti AND ([article]/lim OR [article in press]/lim OR [conference abstract]/lim) AND [embase]/lim

#2: birth control pill: ab,ti AND ([article]/lim OR [article in press]/lim OR [conference abstract]/lim) AND [embase]/lim

#3: 'oral contraceptive agent'/exp

#4: #1 OR #2 OR #3

#5: stroke: ab,ti AND ([article]/lim OR [article in press]/lim OR [conference abstract]/lim) AND [embase]/lim

#6: brain hemorrhage: ab,ti AND ([article]/lim OR [article in press]/lim OR [conference abstract]/lim) AND [embase]/lim

#7: intracerebral hemorrhage: ab,ti AND ([article]/lim OR [article in press]/lim OR [conference abstract]/lim) AND [embase]/lim

#8: cerebral hemorrhage: ab,ti AND ([article]/lim OR [article in press]/lim OR [conference abstract]/lim) AND [embase]/lim

#9: intraparenchymal hemorrhage: ab,ti AND ([article]/lim OR [article in press]/lim OR [conference abstract]/lim) AND [embase]/lim

#10: intracranial hemorrhage: ab,ti AND ([article]/lim OR [article in press]/lim OR [conference abstract]/lim) AND [embase]/lim

#11: cerebral infarction: ab,ti AND ([article]/lim OR [article in press]/lim OR [conference abstract]/lim) AND [embase]/lim

#12: cerebral ischemia: ab,ti AND ([article]/lim OR [article in press]/lim OR [conference abstract]/lim) AND [embase]/lim

#13: brain ischemia: ab,ti AND ([article]/lim OR [article in press]/lim OR [conference abstract]/lim) AND [embase]/lim

#14: cardiovascular disease: ab,ti AND ([article]/lim OR [article in press]/lim OR [conference abstract]/lim) AND [embase]/lim

#15: cerebrovascular disorder: ab,ti AND ([article]/lim OR [article in press]/lim OR [conference abstract]/lim) AND [embase]/lim

#16: cerebrovascular disease: ab,ti AND ([article]/lim OR [article in press]/lim OR [conference abstract]/lim) AND [embase]/lim

#17: subarachnoid hemorrhage: ab,ti AND ([article]/lim OR [article in press]/lim OR [conference abstract]/lim) AND [embase]/lim

#18: #5 OR #6 OR #7 OR #8 OR #9 OR #10 OR #11 OR #12 OR #13 OR #14 OR #15 OR #16 OR #17

#19: #4 AND #18

#20: #4 AND #18 AND [humans]/lim

**S**u**pplemental Table 1. Exclusion reasons**

| **No.** | **Title** | **Year** | **First author** | **Journal** | **Reason** |
| --- | --- | --- | --- | --- | --- |
| 1 | Oral contraception and increased risk of cerebral ischemia or thrombosis | 1973 | CGSS | The New England journal of Medicine | Exposure without eligible quantitative OCPs |
| 2 | Oral contraceptives and stroke in young women. Associated risk factors | 1975 | CGSS | The journal of American Medical Association (JAMA) | Exposure without eligible quantitative OCPs |
| 3 | Use of oral contraceptives, cigarette smoking, and risk of subarachnoid haemorrhage | 1978 | Petitti | Lancet | Exposure without eligible quantitative OCPs |
| 4 | Oral contraceptives and fatal subarachnoid haemorrhage | 1979 | Inman | British medical journal | Exposure without eligible quantitative OCPs |
| 5 | Fatal subarachnoid haemorrhage in young women: role of oral contraceptives | 1981 | Thorogood | British Medical journal (Clinical research ed.) | Exposure without eligible quantitative OCPs |
| 6 | Incidence of arterial disease among oral contraceptive users | 1983 | RCGP | The Journal of the Royal College of General Practitioners | Exposure without eligible quantitative OCPs |
| 7 | Cardiovascular death among women under 40 years of age using low-estrogen oral contraceptives and intrauterine devices in Finland from 1975 to 1984 | 1990 | Hirvonen | American journal of Obstetrics and Gynecology | Exposure without eligible quantitative OCPs |
| 8 | Fatal stroke and use of oral contraceptives: findings from a case-control study | 1992 | Thorogood | American journal of Epidemiology | Exposure without eligible quantitative OCPs |
| 9 | Subarachnoid hemorrhage and hormonal factors in women. A population-based case-control study | 1994 | Longstreth | Annals of internal Medicine | Exposure without eligible quantitative OCPs |
| 10 | The case for a lower dose pill. Assessing the impact of estrogen dose | 1995 | Brown | Organon's magazine on Women & Health | Review |
| 11 | Stroke in users of low-dose oral contraceptives | 1996 | Petitti | The New England journal of Medicine | Exposure without eligible quantitative OCPs |
| 12 | Oral contraception and cerebral thromboembolism | 1997 | Lidegaard | Acta obstetricia et Gynecologica Scandinavica. Supplement | Meeting abstract |
| 13 | Use of low-dose oral contraceptives and stroke in young women | 1997 | Schwartz | Annals of internal Medicine | Exposure without eligible quantitative OCPs |
| 14 | Migraine and stroke in young women: case-control study. The World Health Organisation Collaborative Study of Cardiovascular Disease and Steroid Hormone Contraception | 1999 | Chang | British Medical Journal (BMJ)  (Clinical research ed.) | Outcome less than three quantitative oral contraceptives categories |
| 15 | Association between low-dose oral contraceptive use and stroke in Chinese women | 2002 | Li | Zhonghua Yi Xue Za Zhi | Duplicate report |
| 16 | Ischemic stroke in young women: A nested case-control study using the UK general practice research database | 2004 | Nightingale | Stroke | Exposure without eligible quantitative OCPs |
| 17 | Oral contraceptive use, thrombophilia and their interaction in young women with ischemic stroke | 2006 | Martinelli | Haematologica | Exposure without eligible quantitative OCPs |
| 18 | The contraceptive patch in relation to ischemic stroke and acute myocardial infarction | 2007 | Jick | Pharmacotherapy | Exposure without eligible quantitative OCPs |
| 19 | Inherited thrombophilia and stratification of ischaemic stroke risk among users of oral contraceptives | 2007 | Pezzini | Journal of Neurol Neurosurg Psychiatry | Exposure without eligible quantitative OCPs |
| 20 | Higher risk of venous thrombosis associated with drospirenone-containing oral contraceptives: a population-based cohort study | 2011 | Gronich | Canadian Medical Association journal | Controls not being never-users or non-users |
| 21 | Study on the association of oral contraceptives, angiotensin-converting enzyme gene polymorphisms and risk of stroke in women | 2012 | Li | Zhonghua Liu Xing Bing Xue Za Zhi | Duplicate report |
| 22 | Cardiovascular risk associated with the use of an etonogestrel-containing vaginal ring | 2013 | Dinger | Obstetrics and Gynecology | Exposure without eligible quantitative OCPs |
| 23 | Study on the association of oral contraceptives, angiotensinogen gene polymorphisms and the risk of stroke in women | 2013 | Huang | Zhonghua Liu Xing Bing Xue Za Zhi | Duplicate report |
| 24 | Hormonal contraception and hemorrhagic stroke: A national follow-up study 2001-2010 | 2013 | Lidegaard | European Journal of Contraception and Reproductive Health Care | Meeting abstract |
| 25 | Combined oral contraceptives and risk of pulmonary embolism, stroke and myocardial infarction: A cohort study of 4 million french women | 2013 | Weill | Drug Safety | Meeting abstract |
| 26 | Prevention opportunities for oral contraceptive-associated ischemic stroke | 2014 | Ryan | Stroke | Exposure without eligible quantitative OCPs |
| 27 | Stroke in young adults: Incidence and clinical picture in 280 patients according to their aetiological subtype | 2016 | Arboix | Medicina Clinica | Exposure without eligible quantitative OCPs |
| 28 | Cardiovascular disease incidence among females in South Carolina by type of oral contraceptives, 2000-2013: a retrospective cohort study | 2016 | Samson | Arch Gynecol Obstet | Exposure without eligible quantitative OCPs |
| 29 | Low dose oestrogen combined oral contraception and risk of pulmonary embolism, stroke, and myocardial infarction in five million French women: cohort study | 2016 | Weill | British Medical Journal (BMJ) | Outcome less than three quantitative oral contraceptives categories |

**Abbreviations: OCPs, oral contraceptives.**

**S**u**pplemental Table 2. Characteristics of included studies regarding OCPs use and risk of stroke in women**

| ***Cohort studies*** | | | | | | | | | | |
| --- | --- | --- | --- | --- | --- | --- | --- | --- | --- | --- |
| **Author/**  **Publication year** | **Study Location** | **Study Period** | **Sample Size** | **Age range at baseline**  **(years)** | **Outcome Type** | **Outcome measure** | **Exposure Assessment** | **Stroke Ascertainment** | **OCPs dimension** | **Adjustment Factors** |
| Lidegaard et al.  (2012) | Denmark. | 1995-2009 | 1626158 | 15-49 | IS | First-ever | Medicinal products Registry | National Patients Registry  Causes of Death Registry | Duration; Estrogen  dosage | Age, educational level, calendar year, diabetes, hypertension, hyperlipidemia, arrhythmia, smoking. |
| Gallagher et al.  (2011) | China. | 1989-2000 | 267400 | ≥30 | IS; HS | Fatal | Questionnaire | Cancer and Death Registry | Duration | Age, smoking status. |
| Yang et al.  (2009) | Swedish. | 1991-2004 | 45 699 | 30-49 | IS; HS | First-ever | Questionnaire | National Hospital Discharge Registry | Duration | Age, BMI, education, alcohol drinking, smoking status, pack-years of smoking, physical activity, high blood pressure, and diabetes. |
| Li et al.  (2006) | China. | 1997-2000 | 119638 | 20-65 | IS; HS | First-ever | Interview  Questionnaire | Medical Records | Cessation | Age and residential regions. |
| Beral et al.  (1999) | UK. | 1968-1993 | 46000 | 16-79 | Stroke | Fatal | Medical Records | Medical Records | Duration; Cessation | Age, parity, social class, and smoking. |
| Mant et al.  (1998) | UK. | 1968-1994 | 17032 | 25-39 | IS | First-ever | Interview Questionnaire | Medical Records | Duration; Cessation | Age, social class, smoking, and quetelet index. |
| ***Case-control studies*** | | | | | | | | | | |
| **Author/**  **Publication year** | **Study Location** | **Study Period** | **Cases/ controls** | **Age range at baseline**  **(years)** | **Outcome Type** | **Outcome measure** | **Exposure Assessment** | **Stroke Ascertainment** | **OCPs dimension** | **Adjustment Factors** |
| Wang et al.  (2012) | China. | 1997-2009 | 451/831 | 20-65 | IS; HS | First-ever | Interview  Questionnaire | Medical Records | Duration | Age, region. |
| Siritho et al.  (2003) | Australia. | 1984-1996 | 234/234 | 15-55 | IS | First-ever | Questionnaire | Medical Records | Duration | Age, region, smoking, alcohol consumption, exercise, diabetes, cholesterol, hypertension, myocardial infarction, TIA. |
| Lidegaard et al.  (2002) | Denmark. | 1994-1998 | 626/4054 | 15-44 | IS | First-ever | Questionnaire | National Patients Registry | Duration; Estrogen dosage | Age, year, smoking, migraine, years of schooling, diabetes, hypertension, heart disease, hyperlipidemia, coagulation disturbances, family CTA, family AMI, and family VTE. |
| Kemmeren et al.  (2002) | Netherland. | 1990-1995 | 203/925 | 18-49 | IS | First-ever | Questionnaire | Medical Records | Estrogen dosage | Age, area of residence, calendar year. |
| Heinemann et al.  (1998) | Europe. | 1993-1996 | 220/775 | 16–44 | IS | First-ever | Interview | Medical Records | Duration | Age, smoking status, alcohol use, hypertension, BMI, parity, hypercholesterolemia, family history of stroke, duration of current OCPs use, and diabetes. |
| Lidegaard et al.  (1998) | Denmark. | 1994-1995 | 219/1041 | 15–44 | IS | First-ever | Questionnaire | Medical Records | Duration; Estrogen dosage | Age, smoking, hypertension, diabetes, stress at work, family AMI, years of schooling, heart disease, hyperlipidemia, family stroke, coagulopathies, family VTE, and migraine. |
| Schwartz et al. (1998) | USA. | 1991-1995 | 373/1191 | 18-44 | IS; HS | First-ever | Interview | Medical Records | Duration;  Cessation | Age, hypertension, body mass index, treated diabetes, smoking, menopausal status, and ethnicity. |
| WHO  Study-1  (1996) | Europe and  Developing countries | 1989-1993 | 697/1952 | 20-44 | IS | First-ever | Interview  Questionnaire | Medical Records | Estrogen dosage | EUR: age, number of live-birth,  hypertension, and smoking categories;  DC: age, number of live-birth, smoking categories, rheumatic heart disease, and hypertension. |
| WHO Study-2 (1996) | Europe and Developing countries | 1989-1993 | 1068/2910 | 20-44 | HS | First-ever | Interview  Questionnaire | Medical Records | Estrogen dosage | Age, hypertension and smoking. |
| Tzourio et al.  (1995) | France. | 1990-1993 | 72/173 | 18-44 | IS | First-ever | Interview  Questionnaire | Medical Records | Estrogen dosage | Age, tobacco, smoking, BMI, hypercholesterolaemia, history of hypertension, diabetes, education. |
| Hannaford et al.  (1994) | UK. | 1968-1990 | 253/759 | 21-70 | Stroke | First-ever | Medical Records | Medical Records | Estrogen dosage | Age, smoking and social class. |
| Lidegaard et al.  (1993) | Denmark. | 1985-1991 | 590/1396 | 15-44 | IS | First-ever | Questionnaire | National Patients Registry | Estrogen dosage | Age, smoking, and years of schooling. |

**Abbreviations:** **IS, ischemic stroke; HS, hemorrhagic stroke; BMI, body mass index; TIA, transient ischemic attack; CTA, cerebral thromboembolic attack; AMI, acute myocardial infarction; VTE, venous thromboembolism; OCPs, oral contraceptives; EUR, Europe; DC, developing countries.**

| **Supplemental Table 3. Quality assessment of the included studies by Newcastle–Ottawa Scale** | | | | | | | | | | | | | |
| --- | --- | --- | --- | --- | --- | --- | --- | --- | --- | --- | --- | --- | --- |
| Cohort studies | | | | | | | | | | | | | |
| References | Selection | | | |  | Comparablility | |  | Outcome | | |  | Score |
| (Study, year) | Representative-  ness of exposed cohort | Selection of non-exposed cohort | Ascertainment of exposure | Outcome present at start of study | | Study controls for age | Study controls for any additional important factor | | Assessment of outcome | Length of follow-up | Adequacy of follow-up | |  |
| Lidegaard et al. (2012) | ☆ | ☆ | ☆ |  | | ☆ | ☆ | | ☆ | ☆ | ☆ | | 8 |
| Gallagher et al. (2011) |  | ☆ | ☆ | ☆ | | ☆ | ☆ | | ☆ | ☆ | ☆ | | 8 |
| Yang et al.  (2009) | ☆ | ☆ | ☆ | ☆ | | ☆ | ☆ | | ☆ | ☆ | ☆ | | 9 |
| Li et al.  (2006) | ☆ | ☆ | ☆ | ☆ | | ☆ | ☆ | | ☆ |  | ☆ | | 8 |
| Beral et al.  (1999) | ☆ | ☆ | ☆ | ☆ | | ☆ | ☆ | | ☆ | ☆ |  | | 8 |
| Mant et al.  (1998) | ☆ | ☆ | ☆ | ☆ | | ☆ | ☆ | | ☆ | ☆ | ☆ | | 9 |
| Case-control studies | | | | | | | | | | | | | |
| References | Selection | | | |  | Comparablility | |  | Exposure | | |  | Score |
| (Study, year) | Case definition | Representativ-eness of cases | Control selection | Control definition | | Study controls for age | Study controls for any additional important factor | | Ascertainment of exposure | Same method of ascertainment for cases and controls | Nonresponse rate | |  |
| Wang et al. (2012) | ☆ | ☆ | ☆ | ☆ | | ☆ | ☆ | | ☆ | ☆ |  | | 8 |
| Siritho et al. (2003) | ☆ | ☆ | ☆ | ☆ | | ☆ | ☆ | | ☆ | ☆ |  | | 8 |
| Lidegaard et al. (2002) | ☆ | ☆ | ☆ | ☆ | | ☆ | ☆ | |  | ☆ | ☆ | | 8 |
| Kemmeren et al. (2002) | ☆ | ☆ | ☆ | ☆ | | ☆ | ☆ | |  | ☆ |  | | 7 |
| Heinemann et al. (1998) | ☆ | ☆ | ☆ | ☆ | | ☆ | ☆ | |  | ☆ |  | | 7 |
| Lidegaard et al. (1998) | ☆ | ☆ | ☆ | ☆ | | ☆ | ☆ | |  | ☆ | ☆ | | 8 |
| Schwartz et al. (1998) | ☆ | ☆ | ☆ | ☆ | | ☆ | ☆ | | ☆ | ☆ |  | | 8 |
| WHO Study-1 (1996) | ☆ | ☆ |  | ☆ | | ☆ | ☆ | | ☆ | ☆ |  | | 7 |
| WHO Study-2 (1996) | ☆ | ☆ |  | ☆ | | ☆ | ☆ | | ☆ | ☆ |  | | 7 |
| Tzourio et al. (1995) | ☆ | ☆ |  | ☆ | | ☆ | ☆ | |  | ☆ |  | | 6 |
| Hannaford et al. (1994) | ☆ | ☆ | ☆ | ☆ | | ☆ | ☆ | | ☆ | ☆ |  | | 8 |
| Lidegaard et al. (1993） | ☆ | ☆ | ☆ | ☆ | | ☆ | ☆ | |  | ☆ | ☆ | | 8 |

**Supplemental Table 4. Subgroup analyses regarding OCPs use and risk of stroke in women**

| **Subgroup** | **Ischemic Stroke** | | | | | **Total Stroke** | | | | |
| --- | --- | --- | --- | --- | --- | --- | --- | --- | --- | --- |
|  | **N** | **OR(95%CI)** | **I^2^ (%)** | ***P _heterogeneity_*** | ***P _interaction_*** | **N** | **OR(95%CI)** | **I^2^ (%)** | ***P _heterogeneity_*** | ***P _interaction_*** |
| **Region** |  |  |  |  |  |  |  |  |  |  |
| **Developing countries** |  |  |  |  |  |  |  |  |  |  |
| Duration^a^ | 2 | 1.00(0.70-1.42) | 73.2 | 0.053 | 0.228 | 2 | 1.05(0.84-1.31) | 75.5 | 0.017 | 0.256 |
| Dosage^b^ | 1 | 1.24(1.15-1.32) | / | / | 0.330 | 2 | 1.17(1.05-1.31) | 81.5 | 0.020 | 0.537 |
| **Developed countries** |  |  |  |  |  |  |  |  |  |  |
| Duration^a^ | 8 | 1.32(1.06-1.65) | 85.6 | <0.001 | - | 9 | 1.25(1.05-1.49) | 82.8 | <0.001 | - |
| Dosage^b^ | 7 | 1.19(1.16-1.22) | 0.0 | 0.535 | - | 9 | 1.20(1.16-1.23) | 13.0 | 0.326 | - |
| **Study design type** |  |  |  |  |  |  |  |  |  |  |
| **Prospective** |  |  |  |  |  |  |  |  |  |  |
| Duration^a^ | 4 | 1.09(0.94-0.27) | 49.1 | 0.117 | 0.217 | 5 | 1.12(1.01-1.24) | 45.8 | 0.086 | 0.226 |
| Dosage^b^ | 0 | / | / | / | - | 1 | 1.25(1.13-1.38) | / | / | 0.441 |
| **Retrospective** |  |  |  |  |  |  |  |  |  |  |
| Duration^a^ | 6 | 1.36(1.05-1.77) | 86.0 | <0.001 | - | 6 | 1.30(1.01-1.67) | 86.8 | <0.001 | - |
| Dosage^b^ | 8 | 1.20(1.17-1.22) | 0.0 | 0.518 | - | 8 | 1.19(1.15-1.23) | 35.7 | 0.122 | - |
| **Status of OCPs use** |  |  |  |  |  |  |  |  |  |  |
| **Current** |  |  |  |  |  |  |  |  |  |  |
| Duration^a^ | 4 | 1.65(1.37-1.98) | 58.8 | 0.064 | 0.002 | 4 | 1.65(1.37-1.98) | 58.8 | 0.064 | <0.001 |
| Dosage^b^ | 8 | 1.20(1.17-1.22) | 0.0 | 0.518 | - | 9 | 1.19(1.16-1.23) | 33.8 | 0.128 | - |
| Duration^c^ | 4 | 2.71(1.87-3.93) | 59.2 | 0.061 | 0.002 | 4 | 2.71(1.87-3.93) | 59.2 | 0.061 | <0.001 |
| **Ever** |  |  |  |  |  |  |  |  |  |  |
| Duration^a^ | 6 | 1.06(0.95-1.19) | 35.3 | 0.172 | - | 7 | 1.08(0.98-1.18) | 44.8 | 0.061 | - |
| Dosage^b^ | 0 | / | / | / | - | 0 | / | / | / | - |
| Duration^c^ | 6 | 1.13(0.90-1.41) | 36.1 | 0.166 | - | 7 | 1.16(0.97-1.39) | 42.4 | 0.075 | - |
| **Adjust for alcohol** |  |  |  |  |  |  |  |  |  |  |
| **Yes** |  |  |  |  |  |  |  |  |  |  |
| Duration^a^ | 3 | 1.10(0.93-1.31) | 44.9 | 0.163 | 0.439 | 3 | 1.10(0.97-1.25) | 18.8 | 0.296 | 0.533 |
| Dosage^b^ | 0 | / | / | / | - | 0 | / | / | / | - |
| **NO** |  |  |  |  |  |  |  |  |  |  |
| Duration^a^ | 7 | 1.31(1.02-1.67) | 88.6 | <0.001 | - | 8 | 1.23(1.04-1.46) | 85.2 | <0.001 | - |
| Dosage^b^ | 8 | 1.20(1.17-1.22) | 0.0 | 0.518 | - | 9 | 1.19(1.16-1.23) | 33.8 | 0.128 | - |
| **Adjust for BMI** |  |  |  |  |  |  |  |  |  |  |
| **Yes** |  |  |  |  |  |  |  |  |  |  |
| Duration^a^ | 4 | 1.15(0.95-1.38) | 45.5 | 0.138 | 0.602 | 4 | 1.10(0.97-1.25) | 24.7 | 0.249 | 0.426 |
| Dosage^b^ | 1 | 1.35(1.06-1.73) | / | / | 0.376 | 1 | 1.35(1.06-1.73) | / | / | 0.386 |
| **No** |  |  |  |  |  |  |  |  |  |  |
| Duration^a^ | 6 | 1.29(0.99-1.68) | 90.6 | <0.001 | - | 7 | 1.25(1.03-1.51) | 87.6 | <0.001 | - |
| Dosage^b^ | 7 | 1.20(1.17-1.22) | 0.0 | 0.512 | - | 8 | 1.19(1.15-1.23) | 35.8 | 0.121 | - |
| **Adjust for education** |  |  |  |  |  |  |  |  |  |  |
| **Yes** |  |  |  |  |  |  |  |  |  |  |
| Duration^a^ | 4 | 1.49(1.07-2.08) | 90.0 | <0.001 | 0.087 | 4 | 1.41(1.06-1.88) | 88.4 | <0.001 | 0.054 |
| Dosage^b^ | 5 | 1.19(1.16-1.22) | 0.0 | 0.821 | 0.093 | 5 | 1.19(1.16-1.22) | 0.0 | 0.821 | 0.958 |
| **No** |  |  |  |  |  |  |  |  |  |  |
| Duration^a^ | 6 | 1.10(0.95-1.26) | 48.0 | 0.087 | - | 7 | 1.10(0.98-1.22) | 51.9 | 0.034 | - |
| Dosage^b^ | 3 | 1.26(1.19-1.33) | 0.0 | 0.709 | - | 4 | 1.20(1.13-1.28) | 63.2 | 0.019 | - |
| **Adjust for family stroke-history** |  |  |  |  |  |  |  |  |  |  |
| **Yes** |  |  |  |  |  |  |  |  |  |  |
| Duration^a^ | 3 | 1.51(1.30-1.75) | 0.0 | 0.690 | 0.243 | 3 | 1.51(1.30-1.75) | 0.0 | 0.690 | 0.124 |
| Dosage^b^ | 3 | 1.19(1.13-1.24) | 0.0 | 0.776 | 0.462 | 2 | 1.19(1.13-1.24) | 0.0 | 0.776 | 0.801 |
| **No** |  |  |  |  |  |  |  |  |  |  |
| Duration^a^ | 7 | 1.16(0.92-1.46) | 89.5 | <0.001 | - | 8 | 1.13(0.97-1.32) | 84.1 | <0.001 | - |
| Dosage^b^ | 5 | 1.21(1.17-1.26) | 15.4 | 0.315 | - | 7 | 1.20(1.15-1.25) | 46.8 | 0.059 | - |
| **Adjust for smoking** |  |  |  |  |  |  |  |  |  |  |
| **Yes** |  |  |  |  |  |  |  |  |  |  |
| Duration^a^ | 9 | 1.25(1.01-1.57) | 86.0 | <0.001 | 0.772 | 10 | 1.19(1.01-1.40) | 82.9 | <0.001 | 0.903 |
| Dosage^b^ | 7 | 1.20(1.17-1.22) | 0.0 | 0.511 | 0.376 | 8 | 1.19(1.15-1.23) | 35.4 | 0.124 | 0.388 |
| **No** |  |  |  |  |  |  |  |  |  |  |
| Duration^a^ | 1 | 1.15(1.05-1.27) | / | / | - | 1 | 1.23(1.14-1.32) | / | / | - |
| Dosage^b^ | 1 | 1.29(1.11-1.51) | / | / | - | 1 | 1.29(1.11-1.51) | / | / | - |
| **Adjust for smoking and hypertension** |  |  |  |  |  |  |  |  |  |  |
| **Yes** |  |  |  |  |  |  |  |  |  |  |
| Duration^a^ | 7 | 1.31(1.02-1.67) | 87.6 | <0.001 | 0.412 | 7 | 1.24(1.01-1.53) | 85.9 | <0.001 | 0.442 |
| Dosage^b^ | 5 | 1.20(1.17-1.22) | 2.2 | 0.402 | 0.424 | 6 | 1.18(1.14-1.22) | 43.5 | 0.088 | 0.227 |
| **No** |  |  |  |  |  |  |  |  |  |  |
| Duration^a^ | 3 | 1.10(0.84-1.43) | 60.9 | 0.077 | - | 4 | 1.12(0.97-1.30) | 56.0 | 0.059 | - |
| Dosage^b^ | 3 | 1.24(1.14-1.36) | 0.0 | 0.560 | - | 3 | 1.25(1.17-1.33) | 0.0 | 0.840 | - |

**Abbreviations: OCPs, oral contraceptives; N, number of studies; CI, confidence interval; OR, odds ratio.**

**^a^ for** **every 5-year increment in duration**

**^b^ for** **every 10-µg increment in estrogen dosage**

**^c^ for every 5-year increment in cessation time**

**Supplemental Table 5. Sensitivity analyses regarding OCPs use and risk of stroke**

| **Categories** | **Ischemic Stroke** | | | **Total Stroke** | | |
| --- | --- | --- | --- | --- | --- | --- |
|  | **N** | **OR (95%CI)** | **I^2^ (%)** | **N** | **OR (95%CI)** | **I^2^ (%)** |
| **Random-effects model** |  |  |  |  |  |  |
| Duration^a^ | 10 | 1.24(1.04-1.49) | 85.9 | 11 | 1.20(1.05-1.37) | 81.5 |
| Dosage^b^ | 7 | 1.20(1.17-1.22) | 0.0 | 9 | 1.19(1.16-1.23) | 33.8 |
| Cessation^c^ | 2 | 0.78(0.67- 0.92) | 0.0 | 4 | 0.82(0.68-0.98) | 64.2 |
| **Fixed-effects model** |  |  |  |  |  |  |
| Duration^a^ | 10 | 1.27(1.19-1.35) | 85.9 | 11 | 1.24(1.19-1.31) | 81.5 |
| Dosage^b^ | 7 | 1.20(1.17-1.22) | 0.0 | 9 | 1.19(1.16-1.21) | 33.8 |
| Cessation^c^ | 2 | 0.78(0.67- 0.92) | 0.0 | 4 | 0.84(0.76-0.92) | 64.2 |
| **Analyses within** | | | | | | |
| **Adjust for smoking, hypertension, and diabetes** |  |  |  |  |  |  |
| Duration^a^ | 7 | 1.31(1.02-1.67) | 87.6 | 7 | 1.24(1.01-1.53) | 85.9 |
| Dosage^b^ | 4 | 1.18(1.15-1.21) | 0.0 | 4 | 1.18(1.15-1.21) | 0.0 |
| Cessation^c^ | 1 | 0.79(0.66-0.93) | / | 1 | 0.73(0.63-0.86) | / |
| **Studies where OCPs users refer to non-users** |  |  |  |  |  |  |
| Duration^a^ | 5 | 1.38(1.06-1.79) | 91.2 | 5 | 1.40(1.11-1.77) | 90.2 |
| Dosage^b^ | 5 | 1.20(1.17-1.23) | 2.2 | 6 | 1.18(1.14-1.22) | 44.1 |
| **Subjects include** **age < 18 years** |  |  |  |  |  |  |
| Duration^a^ | 5 | 1.46(1.12-1.90) | 84.2 | 6 | 1.41(1.11-1.78) | 83.6 |
| Dosage^b^ | 4 | 1.18(1.16-1.21) | 0.0 | 4 | 1.18(1.16-1.21) | 0.0 |
| Cessation^c^ | 0 | / | / | 1 | 1.01(0.86-1.18) | / |
| **Analyses without** |  |  |  |  |  |  |
| **Outcome measure (fatal stroke)** |  |  |  |  |  |  |
| Duration^a^ | 9 | 1.30(1.08-1.56) | 86.1 | 9 | 1.26(1.08-1.46) | 83.1 |
| Dosage^b^ | 7 | 1.20(1.17-1.22) | 0.0 | 9 | 1.19(1.16-1.23) | 33.8 |
| Cessation^c^ | 2 | 0.78(0.67- 0.92) | 0.0 | 3 | 0.74(0.66-0.84) | 0.0 |
| **Subjects include age > 50 years** |  |  |  |  |  |  |
| Duration^a^ | 8 | 1.32(1.06-1.65) | 85.6 | 8 | 1.26(1.04-1.53) | 84.2 |
| Dosage^b^ | 8 | 1.20(1.17-1.22) | 0.0 | 8 | 1.19(1.15-1.23) | 35.7 |
| Cessation^c^ | 2 | 0.78(0.67-0.92) | 0.0 | 2 | 0.74(0.65-0.83) | 0.0 |

**^a^ for every 5-year increment in duration**

**^b^ for every 10-µg increment in estrogen dosage**

**^c^ for every 5-year increment in cessation time**

**Supplemental Figure 1. Flowchart of the literature search performed**

## Screening

## Eligibility

## Included

Excluded after reviewing title and abstract

(n = 4766)

Excluded (n = 29)

• Exposure without eligible

• quantitative OCPs (n=19)

• Duplicate report (n=3)

• Meeting abstract (n=3)

• Outcome less than three quantitative

• OCPs categories (n=2)

• Controls not being never-users or

• non-users (n=1)

• Review (n=1)

Records identified through database searching

(n = 5178)

PubMed (n= 4170)

EMBASE (n = 1008)

## Identification

Studies included in quantitative synthesis (meta-analysis)

(n = 18)

Full-text articles assessed for eligibility

(n = 47)

Records screened

(n = 4813)

Records after duplicates removed

(n = 4813)
